# 4-Amino-*N*-(4,6-di­methyl­pyrimidin-2-yl)benzene­sulfonamide–1,4-di­aza­bicyclo­[2.2.2]octane (2/1)

**DOI:** 10.1107/S1600536813027037

**Published:** 2013-10-05

**Authors:** Hadi D. Arman, Trupta Kaulgud, Edward R. T. Tiekink

**Affiliations:** a Department of Chemistry, The University of Texas at San Antonio, One UTSA Circle, San Antonio, Texas 78249-0698, USA; b Department of Chemistry, University of Malaya, 50603 Kuala Lumpur, Malaysia

## Abstract

The asymmetric unit of the title co-crystal, C_12_H_14_N_4_O_2_S·0.5C_6_H_12_N_2_, comprises the sulfonamide mol­ecule and half a mol­ecule of 1,4-di­aza­bicyclo­[2.2.2]octane (DABCO), the latter being disposed about a crystallographic twofold rotation axis. In the sulfonamide mol­ecule, the aromatic rings are almost perpendicular to one another [dihedral angle = 75.01 (8)°]. In the crystal, mol­ecules are connected into a three-mol­ecule aggregate *via* amide–DABCO N—H⋯N hydrogen bonds, and these are connected into a three-dimensional architecture *via* amino–DABCO N—H⋯O and amino-pyrimidine N—H⋯N hydrogen bonds.

## Related literature

For the structure of the sulfonamide, see: Tiwari *et al.* (1984 ▶). For related studies of co-crystal formation, see: Ellis *et al.* (2009 ▶); Arman & Tiekink (2013 ▶). For co-crystals of the sulfonamide with carb­oxy­lic acids, see: Arman *et al.* (2010 ▶); Ghosh *et al.* (2011 ▶); Smith & Wermuth (2013 ▶).
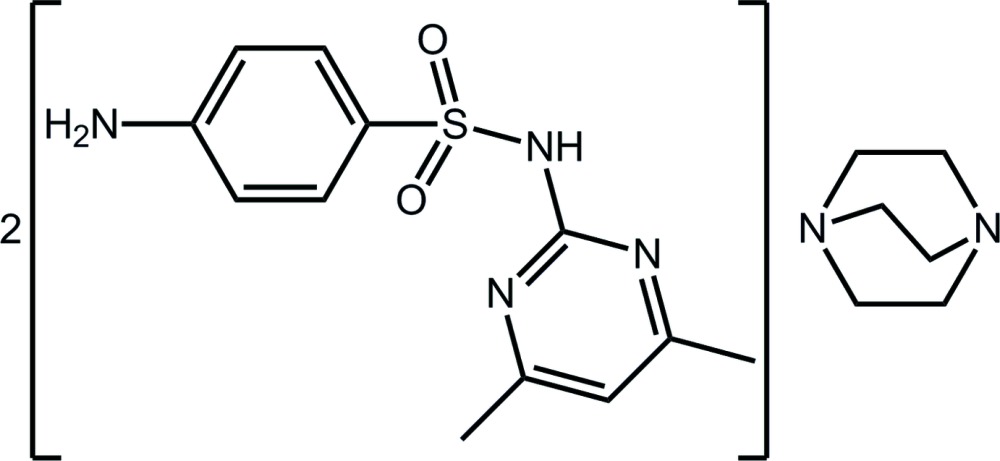


## Experimental

### 

#### Crystal data

C_12_H_14_N_4_O_2_S·0.5C_6_H_12_N_2_
*M* *_r_* = 334.42
Orthorhombic, 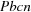

*a* = 26.488 (3) Å
*b* = 9.7886 (11) Å
*c* = 12.2163 (13) Å
*V* = 3167.4 (6) Å^3^
*Z* = 8
Mo *K*α radiation
μ = 0.22 mm^−1^
*T* = 98 K
0.35 × 0.31 × 0.21 mm

#### Data collection

Rigaku AFC12/SATURN724 diffractometer
7965 measured reflections
3616 independent reflections
3264 reflections with *I* > 2σ(*I*)
*R*_int_ = 0.032

#### Refinement

*R*[*F* ^2^ > 2σ(*F* ^2^)] = 0.044
*wR*(*F* ^2^) = 0.115
*S* = 0.99
3616 reflections
219 parameters
3 restraints
H atoms treated by a mixture of independent and constrained refinement
Δρ_max_ = 0.41 e Å^−3^
Δρ_min_ = −0.48 e Å^−3^

### 

Data collection: *CrystalClear* (Molecular Structure Corporation & Rigaku, 2005 ▶); cell refinement: *CrystalClear*; data reduction: *Crystal­Clear*; program(s) used to solve structure: *SHELXS97* (Sheldrick, 2008 ▶); program(s) used to refine structure: *SHELXL97* (Sheldrick, 2008 ▶); molecular graphics: *ORTEP-3 for Windows* (Farrugia, 2012 ▶) and *DIAMOND* (Brandenburg, 2006 ▶); software used to prepare material for publication: *publCIF* (Westrip, 2010 ▶).

## Supplementary Material

Crystal structure: contains datablock(s) general, I. DOI: 10.1107/S1600536813027037/su2652sup1.cif

Structure factors: contains datablock(s) I. DOI: 10.1107/S1600536813027037/su2652Isup2.hkl

Supplementary material file. DOI: 10.1107/S1600536813027037/su2652Isup3.cml

Additional supplementary materials:  crystallographic information; 3D view; checkCIF report

## Figures and Tables

**Table 1:** Hydrogen-bond geometry (Å, °)

*D*—H⋯*A*	*D*—H	H⋯*A*	*D*⋯*A*	*D*—H⋯*A*
N1—H1*N*⋯N5^i^	0.88 (2)	1.90 (2)	2.768 (2)	169 (2)
N4—H2*N*⋯O2^ii^	0.88 (1)	2.48 (2)	3.058 (2)	124 (2)
N4—H2*N*⋯N2^ii^	0.88 (1)	2.59 (2)	3.376 (2)	149 (2)
N4—H3*N*⋯O1^iii^	0.88 (2)	2.15 (2)	3.032 (2)	178 (2)

Symmetry codes: (i) 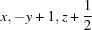; (ii) 
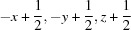
; (iii) 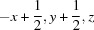.

**Fig. 1.**
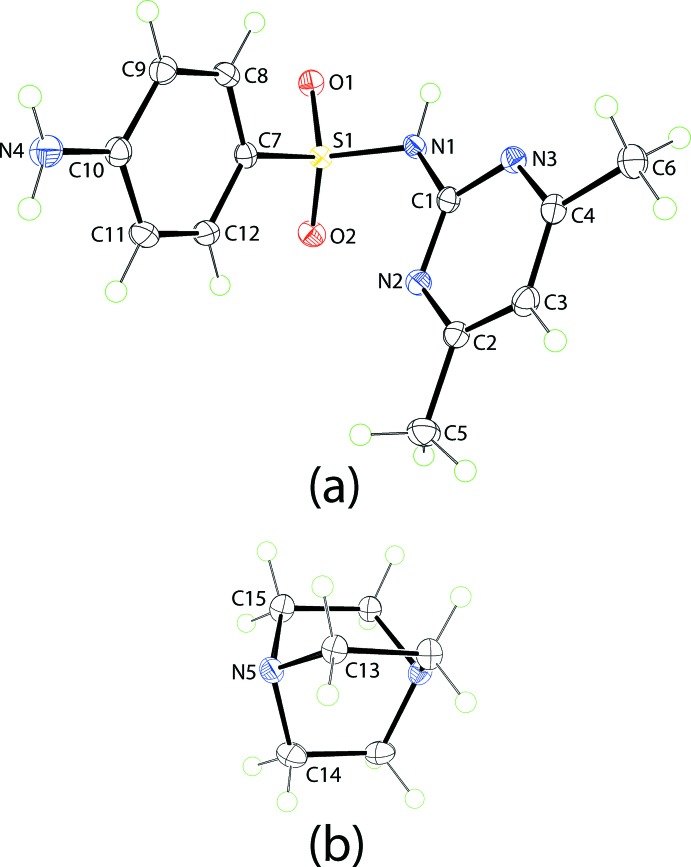
Molecular structures of the components of the title compound, with atom labelling: (a) the sulfonamide and (b) DABCO. Displacement ellipsoids are drawn at the 50% probability level.

**Fig. 2.**
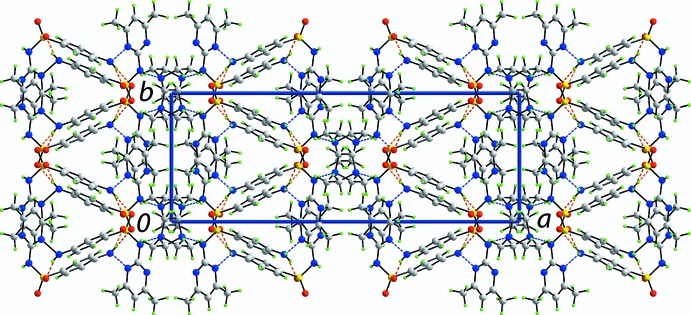
A viewed in projection along the c axis of the crystal packing of the title compound. The N-H···O and N-H···N hydrogen bonds are shown as orange and blue dashed lines, respectively.

## supplementary crystallographic information

### 1. Comment

The title co-crystal was formed in continuation of on-going structural studies of co-crystals (Ellis *et al.*, 2009; Arman & Tiekink, 2013). While co-crystals of the title sulfonamide with carboxylic acids are known (Arman, *et al.* 2010; Ghosh *et al.* 2011; Smith & Wermuth, 2013), the present investigation appears to be the first describing a co-crystal of the sulfonamide with an amine.

The asymmetric unit of the title compound contains a molecule of the sulfonamide in a general position, and half a molecule of 1,4-diazabicyclo[2.2.2]octane (DABCO) which is disposed about a crystallographic two-fold rotation axis, Fig. 1. The overall shape of the sulfonamide approximates the letter *L* with the dihedral angle between the two aromatic rings being 75.01 (8)°, which compares to 78.1 (6)° found in the parent sulfonamide compound (Tiwari *et al.*, 1984). However, this is a little misleading as there is a difference in the conformation of the two sulfonamides. In the title sulfonamide the SOC_6_H_4_NH_2_ residue lies to one side of the pyrimidinyl ring with the remaining O atom, O2, being co-planar giving the *L*-shape, whereas in the parent sulfonamide (Tiwari *et al.*, 1984) the SO_2_ O atoms lie to one side of the pyrimidinyl ring and the C_6_H_4_NH_2_ residue to the other.

A three-dimensional architecture is formed in the crystal structure by N—H···O and N-H···N hydrogen bonds (Fig. 2 and Table 1). The amide group, N1—H1N, forms a hydrogen bond to a DABCO N atom, N5, so that a three-molecule aggregate results. The amino group H atom, N4—H2N, is bifurcated, forming hydrogen bonds to the sulfonamide atom O2 and to the pyrimidinyl atom N2. The second amino group H atom, N4—H3N, forms a hydrogen bond to the second sulfonamide O atom, O1.

### 2. Experimental

Crystals of the title compound were obtained by the co-crystallization of the sulfonamide (ACROS, 0.18 mmol) and 1,4-diazabicyclo[2.2.2]octane (DABCO; Sigma-Aldrich, 0.10 mmol) in methanol. Block-like colourless crystals were obtained by slow evaporation (M.p. = 479–485 K).

### 3. Refinement

The N-bound H-atoms were located in a difference Fourier map and refined with a distance restraint: N—H = 0.88 (1) Å with *U*_iso_(H) = 1.2*U*_eq_(N). The C-bound H-atoms were placed in calculated positions and included in the refinement in the riding model approximation: C—H = 0.95–0.99 Å with *U*_iso_(H) = 1.5*U*_eq_(C-methyl) and = 1.2 U_eq_(C) for other H atoms.

### Crystal data

**Table:**

C_12_H_14_N_4_O_2_S·0.5C_6_H_12_N_2_	*F*(000) = 1416
*M**_r_* = 334.42	*D*_x_ = 1.403 Mg m^−^^3^
Orthorhombic, *P**b**c**n*	Mo *K*α radiation, λ = 0.71073 Å
Hall symbol: -P 2n 2ab	Cell parameters from 11130 reflections
*a* = 26.488 (3) Å	θ = 2.1–40.3°
*b* = 9.7886 (11) Å	µ = 0.22 mm^−^^1^
*c* = 12.2163 (13) Å	*T* = 98 K
*V* = 3167.4 (6) Å^3^	Block, colourless
*Z* = 8	0.35 × 0.31 × 0.21 mm

### Data collection

**Table:**

Rigaku AFC12K/SATURN724 diffractometer	3264 reflections with *I* > 2σ(*I*)
Radiation source: fine-focus sealed tube	*R*_int_ = 0.032
Graphite monochromator	θ_max_ = 27.5°, θ_min_ = 2.2°
ω scans	*h* = −10→34
7965 measured reflections	*k* = −8→12
3616 independent reflections	*l* = −15→10

### Refinement

**Table:**

Refinement on *F*^2^	Primary atom site location: structure-invariant direct methods
Least-squares matrix: full	Secondary atom site location: difference Fourier map
*R*[*F*^2^ > 2σ(*F*^2^)] = 0.044	Hydrogen site location: inferred from neighbouring sites
*wR*(*F*^2^) = 0.115	H atoms treated by a mixture of independent and constrained refinement
*S* = 0.99	*w* = 1/[σ^2^(*F*_o_^2^) + (0.0551*P*)^2^ + 2.7774*P*] where *P* = (*F*_o_^2^ + 2*F*_c_^2^)/3
3616 reflections	(Δ/σ)_max_ < 0.001
219 parameters	Δρ_max_ = 0.41 e Å^−^^3^
3 restraints	Δρ_min_ = −0.48 e Å^−^^3^

### Special details

**Table:**

Geometry. All s.u.'s (except the s.u. in the dihedral angle between two l.s. planes) are estimated using the full covariance matrix. The cell s.u.'s are taken into account individually in the estimation of s.u.'s in distances, angles and torsion angles; correlations between s.u.'s in cell parameters are only used when they are defined by crystal symmetry. An approximate (isotropic) treatment of cell s.u.'s is used for estimating s.u.'s involving l.s. planes.
Refinement. Refinement of *F*^2^ against ALL reflections. The weighted *R*-factor *wR* and goodness of fit *S* are based on *F*^2^, conventional *R*-factors *R* are based on *F*, with *F* set to zero for negative *F*^2^. The threshold expression of *F*^2^ > 2σ(*F*^2^) is used only for calculating *R*-factors(gt) *etc*. and is not relevant to the choice of reflections for refinement. *R*-factors based on *F*^2^ are statistically about twice as large as those based on *F*, and *R*- factors based on ALL data will be even larger.

### Fractional atomic coordinates and isotropic or equivalent isotropic displacement parameters (Å^2^)

**Table:**

	*x*	*y*	*z*	*U*_iso_*/*U*_eq_	
S1	0.132415 (15)	0.06565 (4)	0.45540 (3)	0.01333 (12)	
O1	0.11533 (5)	−0.05810 (12)	0.50942 (10)	0.0173 (3)	
O2	0.13901 (5)	0.05841 (13)	0.33849 (10)	0.0183 (3)	
N1	0.09018 (5)	0.17725 (15)	0.48968 (11)	0.0148 (3)	
H1N	0.0745 (7)	0.152 (2)	0.5494 (11)	0.018*	
N2	0.12538 (5)	0.36203 (15)	0.39075 (11)	0.0160 (3)	
N3	0.07074 (6)	0.39389 (15)	0.54545 (11)	0.0160 (3)	
N4	0.32337 (6)	0.25427 (17)	0.65547 (13)	0.0218 (3)	
H2N	0.3284 (9)	0.250 (2)	0.7267 (8)	0.026*	
H3N	0.3418 (8)	0.308 (2)	0.6139 (16)	0.026*	
C1	0.09647 (6)	0.31690 (17)	0.47297 (13)	0.0148 (3)	
C2	0.12939 (6)	0.49905 (19)	0.38156 (14)	0.0166 (3)	
C3	0.10504 (7)	0.58644 (18)	0.45383 (14)	0.0181 (4)	
H3	0.1085	0.6827	0.4474	0.022*	
C4	0.07545 (7)	0.52934 (18)	0.53588 (14)	0.0169 (3)	
C5	0.16111 (8)	0.55243 (19)	0.28925 (15)	0.0226 (4)	
H5A	0.1968	0.5353	0.3050	0.034*	
H5B	0.1555	0.6509	0.2812	0.034*	
H5C	0.1517	0.5060	0.2212	0.034*	
C6	0.04783 (7)	0.61545 (19)	0.61820 (15)	0.0223 (4)	
H6A	0.0120	0.5909	0.6180	0.033*	
H6B	0.0515	0.7122	0.5990	0.033*	
H6C	0.0620	0.5995	0.6912	0.033*	
C7	0.18946 (6)	0.11773 (17)	0.51417 (13)	0.0144 (3)	
C8	0.19655 (6)	0.10442 (17)	0.62712 (14)	0.0159 (3)	
H8	0.1710	0.0640	0.6712	0.019*	
C9	0.24078 (6)	0.15020 (18)	0.67437 (14)	0.0173 (3)	
H9	0.2456	0.1396	0.7509	0.021*	
C10	0.27886 (7)	0.21239 (18)	0.61103 (14)	0.0172 (3)	
C11	0.27024 (7)	0.2272 (2)	0.49774 (15)	0.0211 (4)	
H11	0.2950	0.2707	0.4535	0.025*	
C12	0.22649 (7)	0.17962 (19)	0.45039 (14)	0.0192 (4)	
H12	0.2216	0.1890	0.3737	0.023*	
N5	0.02978 (6)	0.88272 (15)	0.16824 (11)	0.0165 (3)	
C13	0.05365 (6)	0.80970 (18)	0.26139 (13)	0.0168 (3)	
H13A	0.0862	0.8535	0.2799	0.020*	
H13B	0.0604	0.7137	0.2407	0.020*	
C14	0.01889 (8)	1.02566 (18)	0.20149 (15)	0.0234 (4)	
H14A	0.0043	1.0765	0.1389	0.028*	
H14B	0.0506	1.0716	0.2236	0.028*	
C15	−0.01819 (6)	0.81388 (19)	0.13850 (14)	0.0180 (3)	
H15A	−0.0111	0.7197	0.1133	0.022*	
H15B	−0.0347	0.8639	0.0779	0.022*	

### Atomic displacement parameters (Å^2^)

**Table:**

	*U*^11^	*U*^22^	*U*^33^	*U*^12^	*U*^13^	*U*^23^
S1	0.0140 (2)	0.0121 (2)	0.0139 (2)	−0.00069 (14)	0.00026 (15)	−0.00111 (14)
O1	0.0176 (6)	0.0125 (6)	0.0219 (6)	−0.0016 (5)	0.0005 (5)	0.0000 (5)
O2	0.0206 (6)	0.0201 (6)	0.0142 (6)	−0.0009 (5)	0.0005 (5)	−0.0043 (5)
N1	0.0135 (6)	0.0136 (7)	0.0174 (6)	−0.0002 (5)	0.0036 (5)	0.0003 (5)
N2	0.0162 (7)	0.0152 (7)	0.0166 (7)	0.0004 (5)	0.0013 (6)	0.0005 (6)
N3	0.0162 (7)	0.0162 (7)	0.0157 (6)	0.0013 (6)	0.0006 (5)	−0.0005 (5)
N4	0.0188 (7)	0.0266 (8)	0.0198 (7)	−0.0053 (6)	−0.0043 (6)	0.0018 (6)
C1	0.0123 (7)	0.0162 (8)	0.0158 (7)	0.0002 (6)	−0.0018 (6)	0.0000 (6)
C2	0.0150 (7)	0.0174 (8)	0.0174 (8)	0.0003 (6)	−0.0006 (6)	0.0022 (7)
C3	0.0188 (8)	0.0132 (7)	0.0225 (9)	0.0012 (6)	−0.0007 (7)	0.0011 (6)
C4	0.0158 (8)	0.0172 (8)	0.0176 (8)	0.0027 (6)	−0.0017 (6)	−0.0012 (6)
C5	0.0255 (9)	0.0181 (8)	0.0243 (9)	−0.0014 (7)	0.0057 (8)	0.0028 (7)
C6	0.0246 (9)	0.0188 (8)	0.0234 (9)	0.0047 (7)	0.0034 (7)	−0.0024 (7)
C7	0.0121 (7)	0.0140 (7)	0.0171 (7)	−0.0002 (6)	−0.0002 (6)	−0.0008 (6)
C8	0.0162 (8)	0.0155 (8)	0.0160 (7)	−0.0004 (6)	0.0034 (6)	0.0001 (6)
C9	0.0181 (8)	0.0181 (8)	0.0157 (7)	0.0007 (7)	−0.0015 (6)	−0.0012 (6)
C10	0.0154 (8)	0.0156 (8)	0.0204 (8)	−0.0002 (6)	−0.0008 (7)	0.0006 (7)
C11	0.0163 (8)	0.0255 (9)	0.0214 (8)	−0.0039 (7)	0.0018 (7)	0.0064 (7)
C12	0.0154 (8)	0.0251 (9)	0.0172 (8)	−0.0008 (7)	−0.0005 (7)	0.0053 (7)
N5	0.0166 (7)	0.0174 (7)	0.0154 (6)	−0.0021 (6)	0.0007 (5)	0.0016 (6)
C13	0.0151 (8)	0.0194 (8)	0.0159 (7)	0.0004 (6)	−0.0001 (6)	−0.0003 (6)
C14	0.0300 (10)	0.0144 (8)	0.0258 (9)	−0.0003 (7)	0.0000 (8)	0.0028 (7)
C15	0.0141 (7)	0.0234 (9)	0.0165 (7)	−0.0010 (7)	−0.0009 (6)	−0.0013 (7)

### Geometric parameters (Å, º)

**Table:**

S1—O2	1.4406 (12)	C6—H6C	0.9800
S1—O1	1.4518 (12)	C7—C12	1.392 (2)
S1—N1	1.6188 (14)	C7—C8	1.399 (2)
S1—C7	1.7488 (17)	C8—C9	1.381 (2)
N1—C1	1.392 (2)	C8—H8	0.9500
N1—H1N	0.876 (9)	C9—C10	1.409 (2)
N2—C1	1.338 (2)	C9—H9	0.9500
N2—C2	1.350 (2)	C10—C11	1.410 (2)
N3—C4	1.337 (2)	C11—C12	1.377 (2)
N3—C1	1.348 (2)	C11—H11	0.9500
N4—C10	1.361 (2)	C12—H12	0.9500
N4—H2N	0.881 (9)	N5—C15	1.483 (2)
N4—H3N	0.877 (10)	N5—C14	1.485 (2)
C2—C3	1.388 (2)	N5—C13	1.485 (2)
C2—C5	1.500 (2)	C13—C15^i^	1.542 (2)
C3—C4	1.390 (2)	C13—H13A	0.9900
C3—H3	0.9500	C13—H13B	0.9900
C4—C6	1.502 (2)	C14—C14^i^	1.551 (4)
C5—H5A	0.9800	C14—H14A	0.9900
C5—H5B	0.9800	C14—H14B	0.9900
C5—H5C	0.9800	C15—C13^i^	1.542 (2)
C6—H6A	0.9800	C15—H15A	0.9900
C6—H6B	0.9800	C15—H15B	0.9900
			
O2—S1—O1	116.57 (7)	C12—C7—S1	120.40 (13)
O2—S1—N1	111.93 (8)	C8—C7—S1	119.60 (13)
O1—S1—N1	103.30 (7)	C9—C8—C7	119.74 (16)
O2—S1—C7	108.46 (8)	C9—C8—H8	120.1
O1—S1—C7	109.02 (8)	C7—C8—H8	120.1
N1—S1—C7	107.11 (8)	C8—C9—C10	121.19 (15)
C1—N1—S1	122.82 (12)	C8—C9—H9	119.4
C1—N1—H1N	117.0 (14)	C10—C9—H9	119.4
S1—N1—H1N	110.8 (14)	N4—C10—C11	120.04 (16)
C1—N2—C2	115.81 (15)	N4—C10—C9	122.07 (16)
C4—N3—C1	116.74 (15)	C11—C10—C9	117.86 (16)
C10—N4—H2N	120.7 (15)	C12—C11—C10	120.92 (16)
C10—N4—H3N	115.6 (15)	C12—C11—H11	119.5
H2N—N4—H3N	121 (2)	C10—C11—H11	119.5
N2—C1—N3	126.72 (16)	C11—C12—C7	120.37 (16)
N2—C1—N1	120.18 (15)	C11—C12—H12	119.8
N3—C1—N1	113.09 (15)	C7—C12—H12	119.8
N2—C2—C3	121.51 (16)	C15—N5—C14	109.18 (14)
N2—C2—C5	116.91 (15)	C15—N5—C13	109.49 (13)
C3—C2—C5	121.58 (16)	C14—N5—C13	109.06 (13)
C2—C3—C4	118.22 (16)	N5—C13—C15^i^	109.61 (13)
C2—C3—H3	120.9	N5—C13—H13A	109.7
C4—C3—H3	120.9	C15^i^—C13—H13A	109.7
N3—C4—C3	120.97 (16)	N5—C13—H13B	109.7
N3—C4—C6	116.90 (16)	C15^i^—C13—H13B	109.7
C3—C4—C6	122.13 (16)	H13A—C13—H13B	108.2
C2—C5—H5A	109.5	N5—C14—C14^i^	109.52 (9)
C2—C5—H5B	109.5	N5—C14—H14A	109.8
H5A—C5—H5B	109.5	C14^i^—C14—H14A	109.8
C2—C5—H5C	109.5	N5—C14—H14B	109.8
H5A—C5—H5C	109.5	C14^i^—C14—H14B	109.8
H5B—C5—H5C	109.5	H14A—C14—H14B	108.2
C4—C6—H6A	109.5	N5—C15—C13^i^	109.84 (13)
C4—C6—H6B	109.5	N5—C15—H15A	109.7
H6A—C6—H6B	109.5	C13^i^—C15—H15A	109.7
C4—C6—H6C	109.5	N5—C15—H15B	109.7
H6A—C6—H6C	109.5	C13^i^—C15—H15B	109.7
H6B—C6—H6C	109.5	H15A—C15—H15B	108.2
C12—C7—C8	119.89 (15)		
			
O2—S1—N1—C1	−66.25 (15)	O2—S1—C7—C8	−166.16 (13)
O1—S1—N1—C1	167.54 (13)	O1—S1—C7—C8	−38.28 (16)
C7—S1—N1—C1	52.51 (15)	N1—S1—C7—C8	72.86 (15)
C2—N2—C1—N3	1.0 (3)	C12—C7—C8—C9	−1.4 (3)
C2—N2—C1—N1	−179.64 (15)	S1—C7—C8—C9	−177.55 (13)
C4—N3—C1—N2	−1.6 (3)	C7—C8—C9—C10	1.0 (3)
C4—N3—C1—N1	179.01 (15)	C8—C9—C10—N4	−177.89 (17)
S1—N1—C1—N2	28.3 (2)	C8—C9—C10—C11	0.4 (3)
S1—N1—C1—N3	−152.26 (12)	N4—C10—C11—C12	176.87 (18)
C1—N2—C2—C3	0.3 (2)	C9—C10—C11—C12	−1.5 (3)
C1—N2—C2—C5	−179.32 (15)	C10—C11—C12—C7	1.1 (3)
N2—C2—C3—C4	−1.0 (3)	C8—C7—C12—C11	0.3 (3)
C5—C2—C3—C4	178.66 (16)	S1—C7—C12—C11	176.48 (15)
C1—N3—C4—C3	0.8 (2)	C15—N5—C13—C15^i^	−61.24 (15)
C1—N3—C4—C6	−178.58 (15)	C14—N5—C13—C15^i^	58.17 (18)
C2—C3—C4—N3	0.4 (3)	C15—N5—C14—C14^i^	57.7 (2)
C2—C3—C4—C6	179.74 (16)	C13—N5—C14—C14^i^	−61.9 (2)
O2—S1—C7—C12	17.69 (17)	C14—N5—C15—C13^i^	−61.68 (17)
O1—S1—C7—C12	145.57 (14)	C13—N5—C15—C13^i^	57.66 (16)
N1—S1—C7—C12	−103.29 (15)		

Symmetry code: (i) −*x*, *y*, −*z*+1/2.

### Hydrogen-bond geometry (Å, º)

**Table:**

*D*—H···*A*	*D*—H	H···*A*	*D*···*A*	*D*—H···*A*
N1—H1*N*···N5^ii^	0.88 (2)	1.90 (2)	2.768 (2)	169 (2)
N4—H2*N*···O2^iii^	0.88 (1)	2.48 (2)	3.058 (2)	124 (2)
N4—H2*N*···N2^iii^	0.88 (1)	2.59 (2)	3.376 (2)	149 (2)
N4—H3*N*···O1^iv^	0.88 (2)	2.15 (2)	3.032 (2)	178 (2)

Symmetry codes: (ii) *x*, −*y*+1, *z*+1/2; (iii) −*x*+1/2, −*y*+1/2, *z*+1/2; (iv) −*x*+1/2, *y*+1/2, *z*.
